# ChillsDB 2.0: Individual Differences in Aesthetic Chills Among 2,900+ Southern California Participants

**DOI:** 10.1038/s41597-023-02816-6

**Published:** 2023-12-21

**Authors:** Felix Schoeller, Leo Christov Moore, Caite Lynch, Nicco Reggente

**Affiliations:** 1 Institute for Advanced Consciousness Studies, Santa Monica, California USA; 2 Massachusetts Institute of Technology, Media Lab, Cambridge, USA

**Keywords:** Motivation, Human behaviour, Consciousness

## Abstract

We significantly enriched ChillsDB, a dataset of audiovisual stimuli validated to elicit aesthetic chills. A total of 2,937 participants from Southern California were exposed to 40 stimuli, consisting of 20 stimuli (10 from ChillsDB and 10 new) presented either in audiovisual or audio-only formats. Questionnaires were administered assessing demographics, personality traits, state affect, and political orientation. Detailed data on chills responses is captured alongside participants’ ratings of the stimuli. The dataset combines controlled elicitation of chills using previously validated materials with individual difference measures to enable investigation of predictors and correlates of aesthetic chills phenomena. It aims to support continued research on the mechanisms and therapeutic potential of aesthetic chills responses.

## Background & Summary

Aesthetic chills are a universal marker of human peak experiences across domains and cultures^1–3^. Characterized by goosebumps and cold shivers down the spine, chills are psychogenic bodily reactions triggered by engaging with evocative stimuli like music or stories^2,4,5^. As a conscious, measurable emotion with neural and behavioral correlates, chills show promise for elucidating the relationship between physiology and affect^1,6,7^, and for the enhancing positive affect in clinically relevant populations^8,9^. Studies indicate chills increase altruism, pleasure, attention and memory^10,11^, modulate heart rate, pupils, skin conductance and muscle contractions^12–14^, and can generate positive shift in mood and emotion in depression^8^. Despite the richness of the phenomenon, most research has been limited to music as the primary stimulus^6,7,10^.

Several databases of stimuli exist to elicit emotions in the laboratory (see review in Table 1). However, none of these databases is focused exclusively on chills making it difficult for researchers interested in this phenomenon to reliably induce the emotion in populations of interest. To fill this gap, ChillsDB^15^ was designed to offer a validated database of audiovisual aesthetic chills stimuli using a novel approach of mining social media content. The original ChillsDB tested 204 potential stimuli across 600 participants. In the current iteration, data was collected from a significantly larger sample of 2,937 participants, all based in Southern California, and balanced for an even representation of political orientation, sex, age and education level. Participants were presented with 20 stimuli, consisting of a subset of 10 stimuli drawn from the previously validated set as well as 10 new stimuli. A total of 20 stimuli were presented to participants in two formats-audio only and audiovisual-resulting in 40 total stimulus presentations with each format represented equally. Additional participant data was collected concerning demographics, personality trait dispositions, and political orientation. Stimulus-related data included pre- and post-stimulus state affect, valence, and mood ratings as well as questionnaires aimed at characterizing post-stimulus state phenomenology. This expansion of the dataset allows for a more in-depth exploration of trait, state and demographic factors affecting aesthetic chills, as well as associated phenomenology Fig. 2.

**Table 1:** Prior work of databases of emotion elicitation stimuli.

DB	Stimuli type	N Participants	N Stimuli
DEAP: The Database for Emotion Analysis Using Physiological Signals^30^	film, music video	40	32
MAAFS: Moral and Affective Film Set^31^	film, YouTube	344	322 American and 253 Australian participants
Dataset of professional and amateur videos that elicit basic emotions^32^	film, pro videos and amateur videos (YT)	40	30
The Child Emotion Facial Expression Set^33^	film	3,668	2 experts
Padova Emotional Dataset of Facial Expressions (PEDFE)^34^	film, facial expression clips	1458	122
CAAV: Chieti Affective Action Videos^35^	film, actions	90 filmed in 4 different versions (perspective, gender)	444
MAHNOB–HCI database^?^	film	20	30
DECAF database^36^	film	40 music videos, 36 movie clips	30
FilmStim database^37^	film	70	364
MAHNOB–laughter	film	nan	22
Film Library for Affective Scientist^38^	film	300	411

## Methods

### Database design

We selected 40 stimuli (20 audio and 20 audiovisual) combining a subset from the original Chills DB (N = 10) and a novel subset obtained from additional parsing using the ChillsDB method and internal polling (N = 10). Each of the 20 stimuli was presented to participants in either two formats - audiovisual or audio-only - in order to compare and test for differences between the two presentation modalities. ChillsDB stimuli were harvested from online social media platforms, YouTube and Reddit, using a Python-based tool to find stimuli distributed across social media platforms using breadth-first search algorithm^16^ (textcolorredNote that all the stimuli used in this experiment were extracted from YouTube). To validate the stimulus set, we used the Qualtrics online platform to recruit participants. For more details on harvesting, refer to the original ChillsDB article^15^.

### Participants

A total of 3,259 participants initially took part in the experiment. Participants were recruited and compensated through Qualtrics. Recruitment was performed according to the following quotas: southern california residents only, approximately equal gender distribution (though gender fluid, nonbinary, and non reporting respondents were also included), and racial/ethnic distributions approximately conforming to southern california census data. Participants in this study were compensated the equivalent of $12 per hour for their participation in the study. Given initial piloting suggesting an average of 20–30 minutes for completion, all participants were compensated $8 for their responses to ensure sufficient, consistent compensation. Payment was made via the Qualtrics platform as follows: Panelists join from a variety of sources. They may be airline customers who chose to join in reward for SkyMiles, retail customers who opted in to get points at their favorite retail outlet, or general consumers who participate for cash or gift cards, etc. When participants are invited to take a survey, they are informed what they will be compensated before they enter the survey. We assessed the data validity by adding qualitative questions (“If you experienced chills, please explain what in the video gave you chills and why you think that is”). These questions were carefully reviewed by two experts and subjects whose response were incoherent with their report were deleted from the dataset. Following this initial check, we identified 932 participants who reported a non-zero Chills Intensity (Mean I = 19.6; SD = 25) despite reporting that they did not experience chills. This was further corroborated in their qualitative descriptions, where the majority stated explicitly they did not experience chills. We eliminated all instances (N = 219) where Chills Intensity exceeded 1 standard deviation from the mean (Mean I = 10.3, SD = 20.7). We retained only those participants (N = 656) whose qualitative responses unambiguously confirmed the absence of chills. These participants were placed in the ‘No Chills’ category and were subsequently omitted from the Chills Intensity analysis. A small number (N) of subjects were eliminated who reported 0 chills intensity despite reporting chills as this indicated an unreliable responder. Following these data cleaning procedures, the experiment involved a diverse group of 2,937 participants, all of whom hailed from Southern California (see Tables 2, 3 for the age and education distributions). The gender distribution was fairly balanced, with 54.24% identifying as female and 41.44% as male. In terms of political affiliation, the largest group identified as Democrats (50.66%), followed by Republicans (21.59%), and Independents (14.81%). Notably, 11.64% of participants did not specify a political affiliation, while a small proportion (1.19%) identified with other political affiliations. Political Orientation was probed as well and is reported in the database. In regards to racial identity, the majority of participants (68.44%) identified as White or Caucasian. The second largest racial group was those identifying as Other (11.37%), followed by American Indian/Native American or Alaska Native (4.97%), and Black or African American (1.46%). A small percentage of participants (0.31%) identified as Asian. Given this broad demographic range, the present dataset provides a rich, representative sample for examining the phenomena under investigation in the context of Southern California.

**Table 2:** Table A1: Distribution of Participants by Age.

	Percentage
65+	22.23%
35–44	20.94%
25–34	17.53%
18–24	13.52%
45–54	12.90%
55–64	12.87%

**Table 3:** Table A2: Distribution of Participants by Education Level.

	Percentage
Bachelor’s degree	24.96%
Some college, but no degree	23.19%
Graduate or professional degree (MA, MS, MBA, PhD, JD, MD, DDS, etc.)	21.18%
High school diploma or GED	15.63%
Associates or technical degree	12.16%
Some high school or less	2.45%
Prefer not to say	0.44%

### Ethics

The experiment was conducted in compliance with the Helsinki Declaration. The protocol was granted an exemption status (Advarra IRB Exemption Pro00068209). All participants gave their voluntary informed consent and procedures followed the Ethics Code of the American Psychological Association. All participants were informed about the purpose of the research, their right to decline to participate and to withdraw from the experiment, and the limits of confidentiality. We also provided them with a contact for any questions concerning the research and with the opportunity to ask any questions regarding the phenomenon under study (aesthetic chills) and receive appropriate answers.

## Data Records

### Dataset structure

The ChillsDB 2.0. dataset is released under a Creative Commons Attribution 4.0 International (CC BY 4.0) license on FigShare^17^. This allows others to freely share and adapt the dataset as long as appropriate credit is given to the original creators by citing the published paper (Schoeller *et al*., 2023). The dataset is divided into two .csv files available under a CC BY 4.0 license on the associated FigShare^17^. For a comprehensive understanding of each column, researchers are advised to refer to the **Header Explanation File** 5 (see Table 5).

1. **Data File**: This file contains the primary data collected from participants.
2. **Traits Questionnaires File**: This file contains the full questionnaires assessing personality traits including DPES, MODTAS, KAMF, and NEO-FFI that were completed by participants.
3. **Header Explanation File**: This file provides explanations and more detailed descriptions for each of the columns in the data file.
4. **Stimuli File**: This file provides explanations and URL to the stimuli on a private YouTube channel (contact the authors for the.mp4).

#### Stimuli

**Fig. 1:**
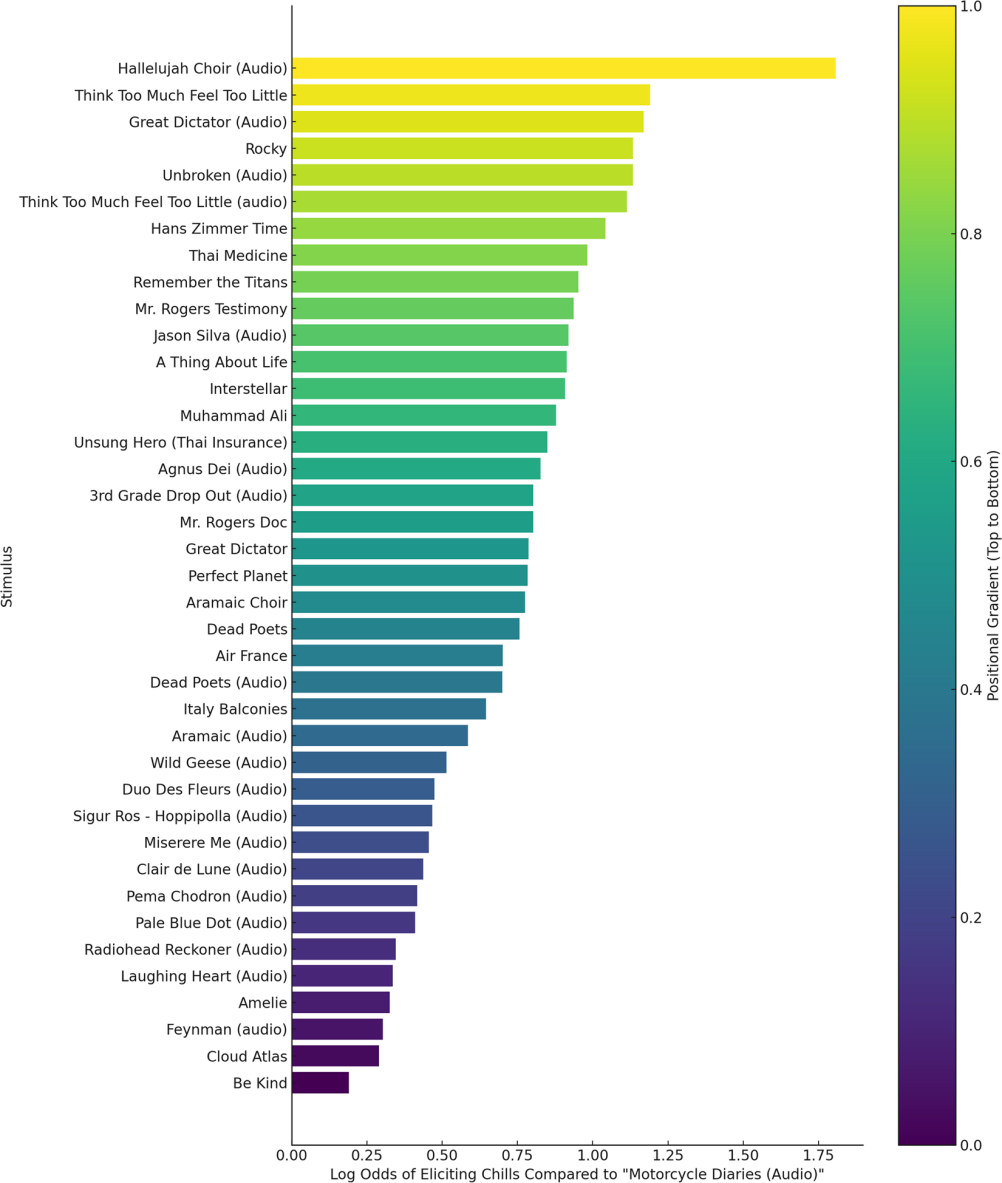
Stimuli Probability of Eliciting Chills: The bar chart illustrates the log odds of each stimulus eliciting chills when compared to “Motorcycle Diaries (Audio)” (the lowest chills ratio stimulus), which is used as the reference stimulus. The gradient color represents a visual ranking from top to bottom, with stimuli at the top having the highest probability of eliciting chills.

The stimuli are listed in the Stimuli.csv file in the dataset (see also Table 4 and Fig. 1). Due to copyright reasons, all stimuli were stored on a private server. Please contact the authors to be granted access to the.mp4 of the stimuli. We curated 40 stimuli (20 audio and 20 audiovisual), of which 10 each were directly sourced from the original Chills DB. To compare the efficacy of individual expert recommendations against crowd-sourced ones, we solicited individual suggestions from the study authors and their extended social networks regarding potential stimuli eliciting aesthetic chills. We then identified overlaps in these recommendations, which were selected to complete the remaining 10 (N = 10) stimuli for our study.

**Table 4:** Top 10 Stimuli with Chills Ratio (N≥70).

Stimulus	Chills ratio
Hallelujah Choir (Audio)	75.7%
Think Too Much Feel Too Little	62.7%
Great Dictator (Audio)	62.2%
Rocky	61.3%
Unbroken (Audio)	61.3%
Think Too Much Feel Too Little (audio)	60.9%
Hans Zimmer Time	59.2%
Thai Medicine	57.7%
Remember the Titans	57.0%
Mr. Rogers Testimony	56.6%

**Fig. 2:**
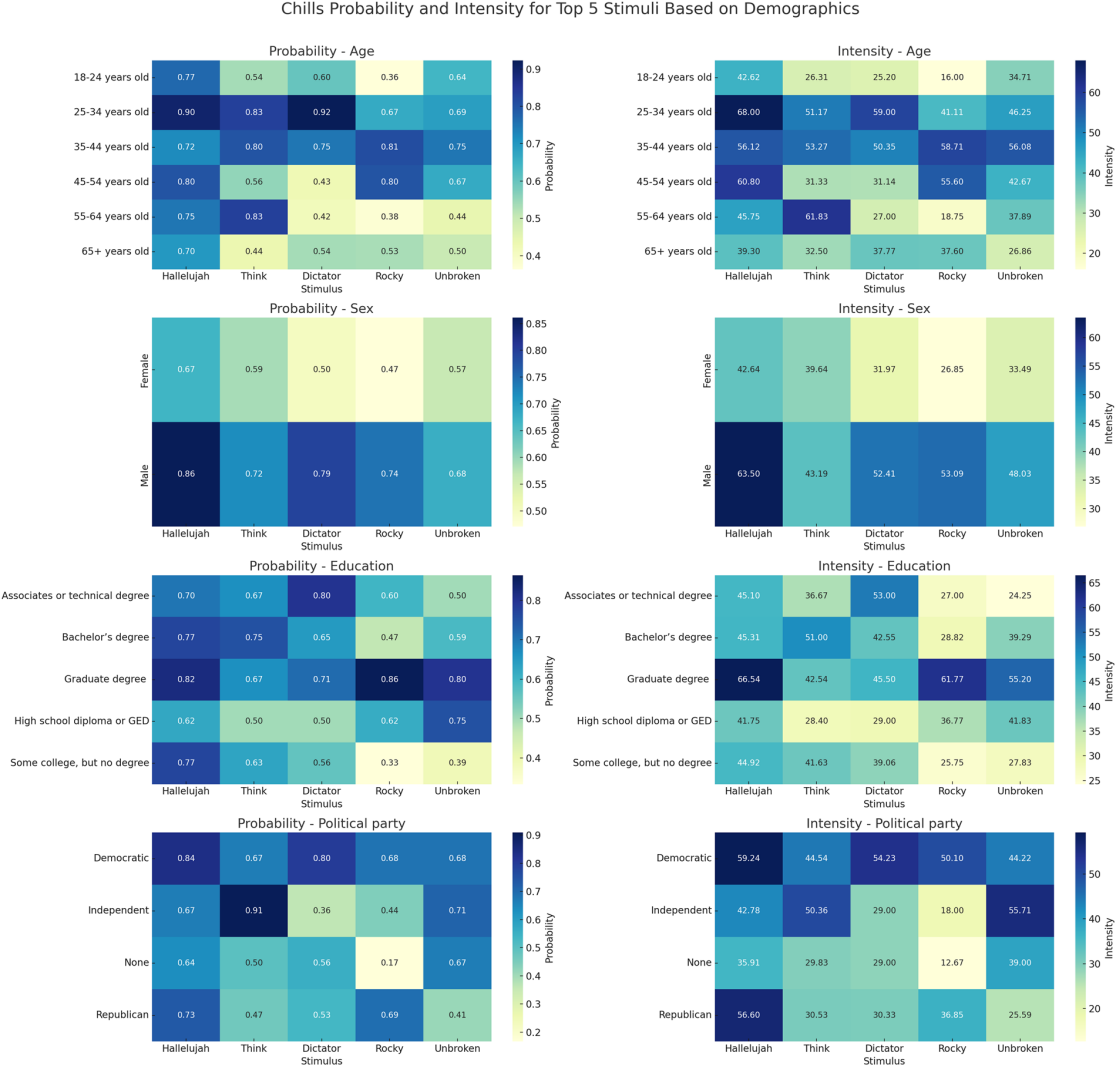
The figure presents heatmaps showcasing the probability and intensity of chills experienced by participants when exposed to different stimuli, segmented by demographic variables such as Age, Sex, Education, and Political Party. Among the striking observations, the stimulus “Hallelujah” profoundly resonates with participants holding a graduate or professional degree, showing both a high probability and intensity of chills. Similarly, individuals within the age bracket of 25–34 years exhibit a pronounced response to “Hallelujah”. This suggests that certain stimuli have a potent emotional impact on specific demographic groups, highlighting the intricate interplay between sensory experiences and personal or cultural backgrounds.

## Technical Validation

Participants were recruited for this study through an online platform (Qualtrics.com) with a focus on individuals residing in Southern California (see Participants section). Before proceeding with the study, participants underwent an initial screening to confirm their geographical location and provided their informed consent. Participants were then asked to provide basic demographic information including gender, education level, and age. Additionally, participants were queried about their political orientation and whether they were affiliated with any political party. In order to assess participants’ affective state, they were prompted to indicate their levels of valence and arousal. Subsequently, participants completed trait questionnaires, including the *Disposition Positive Affect (DEPS)*^18^, *NEO Five-Factor Inventory (NEOFFI)*^19^, *Modified Tellegen Absorption Scale (MODTAS)*^20^, and *Kama Muta Questionnaire (KAMF)*^21^. Participants were then randomly assigned to one of 40 stimulus conditions. After exposure to the assigned stimulus, participants were asked to report their emotional state in terms of valence and arousal once again. They were also asked to indicate how much they liked the video, whether they had seen the video previously, whether they experienced chills while watching the video, and if so, to rate the frequency and intensity of their chills. Participants were also asked whether the video reminded them of a personal experience, and if they experienced goosebumps or tears, they were asked to indicate what elicited those responses. Following the assessment of participants’ immediate responses to the stimulus, they were directed to complete a set of state questionnaires including the *Watts Connectedness Scale*^22^, *Ego Dissolution Scale*^23^, and *Kama Muta Scale*^24^. Upon completion of the study, participants were thanked for their participation and provided with appropriate remuneration for their time and effort. The average duration of each experiment was approximately 40 minutes.

## Usage Notes

The ChillsDB 2.0 database builds upon the foundation established by the original ChillsDB, offering an extended scope for aesthetic chills research. Identifying and validating stimuli that can robustly elicit positive affective states as well as phenomenological states such as ego-dissolution, connectedness and moral elevation, is of value to cognitive and affective neuroscience. Being able to control and manipulate these stimuli in a laboratory setting provides researchers the experimental control needed to map precise relationships between neural activity and phenomenology. Without standardized, validated stimuli capable of provoking robust and measurable affective reactions under controlled conditions, researchers lack a solid basis for elucidating the complex neurobiology underlying human emotion and peak experiences. Indeed, future research should further validate these stimuli by including objective physiological measures in addition to the subjective report that formed the basis of our analysis Table 5.

**Table 5:** Key columns in the Data File.

Column Name	Description
ID	Unique identifier for each participant.
Chills?	Did the participant experience chills?
Goosebumps	Did the participant experience goosebumps?
Stimulus	The stimulus presented to the participant.
Age	Age of the participant.
Sex	Gender of the participant.
Education	Educational background of the participant.
Ethnicity	Ethnic background of the participant.
Political preferences	Political orientation of the participant.
Arousal Pre/Post	Arousal level before and after stimulus exposure.
Valence Pre/Post	Valence rating (positive/negative feeling) before and after exposure.
Liking	How much the participant liked the stimulus.

While the initial ChillsDB was centered on identifying and validating chills-eliciting content, ChillsDB 2.0 provides a more comprehensive perspective via a rich set of trait predictors and state correlates 2. Notably, a leading stimulus in this updated database demonstrates a 0.75 probability of inducing chills across over 70 participants. This database may also have clinical relevance where this expanded database can contribute to research in areas like depression, where aesthetic chills have been shown to mitigate maladaptive cognition^25^, improve hedonic tone^26^, and aberrant chills response may be a physiological signature of anhedonia^8^. Chills are also a key target for the development of body-based, interoceptive technologies enhancing the distinct somatic markers of the emotion^9,27^. At the social scale, deep analysis of chills-eliciting materials could elucidate themes and narratives centrally important to human meaning-making^3,28^. The stimuli that reliably elicit aesthetic chills, as catalogued here, may tap into content that resonates deeply for individuals and cultures^?^. With a larger participant sample and detailed individual difference measures, ChillsDB 2.0 may facilitate a deeper understanding of the factors influencing chills experiences, potentially bridging neuroaesthetics and broader psychological research^29^.

## Data Availability

The code for parsing YouTube and Reddit networks is available under an MIT license at https://github.com/ChillsTV/AffectiveStimuliScraper.
